# Supplementary figures and images for: Dissociation of the nuclear basket triggers chromosome loss in aging yeast
Source: eLife. 2025 Oct 30;14:RP104530. doi: 10.7554/eLife.104530 (PMC12574999; doi:10.7554/eLife.104530)

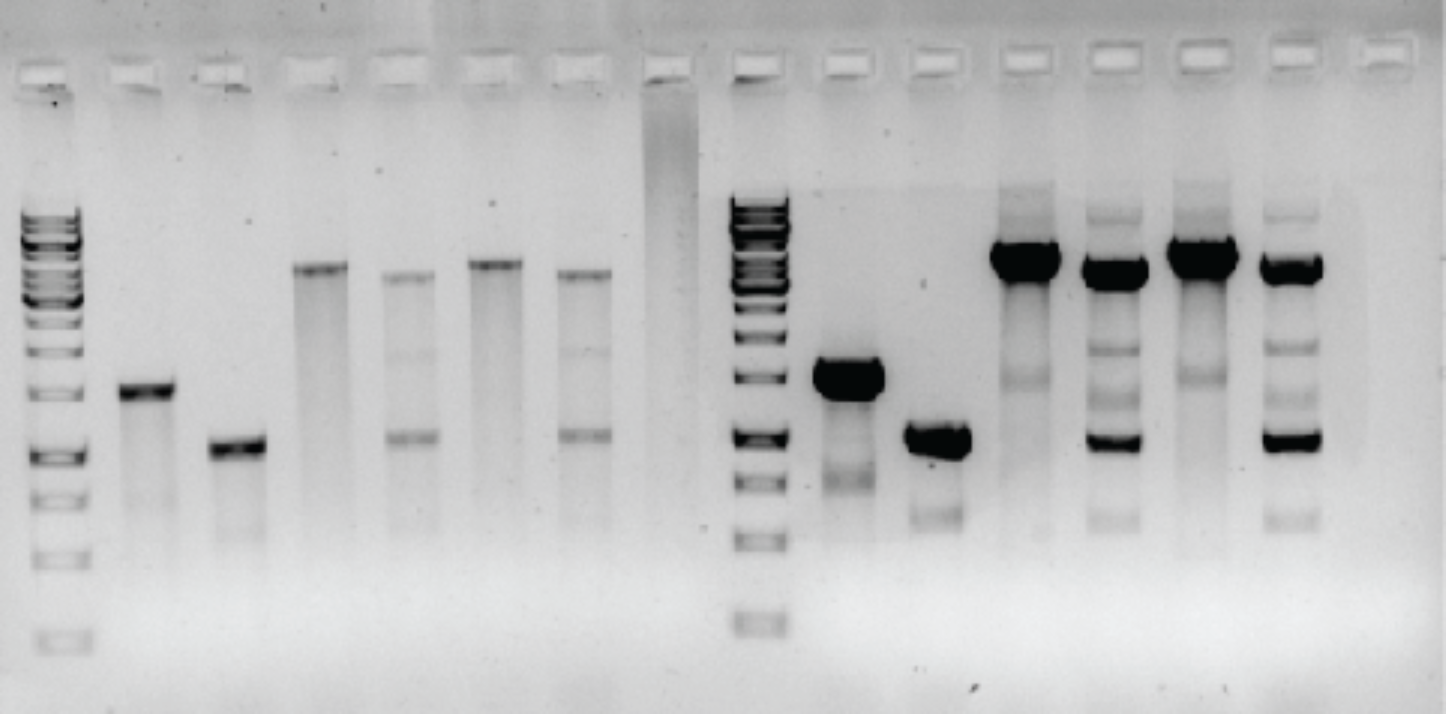

Supplement: Figure 4—figure supplement 1—source data 1. [file elife-104530-fig4-figsupp1-data1.zip › Figure4-S1-source data1/PCR uncroped no mark.tif]

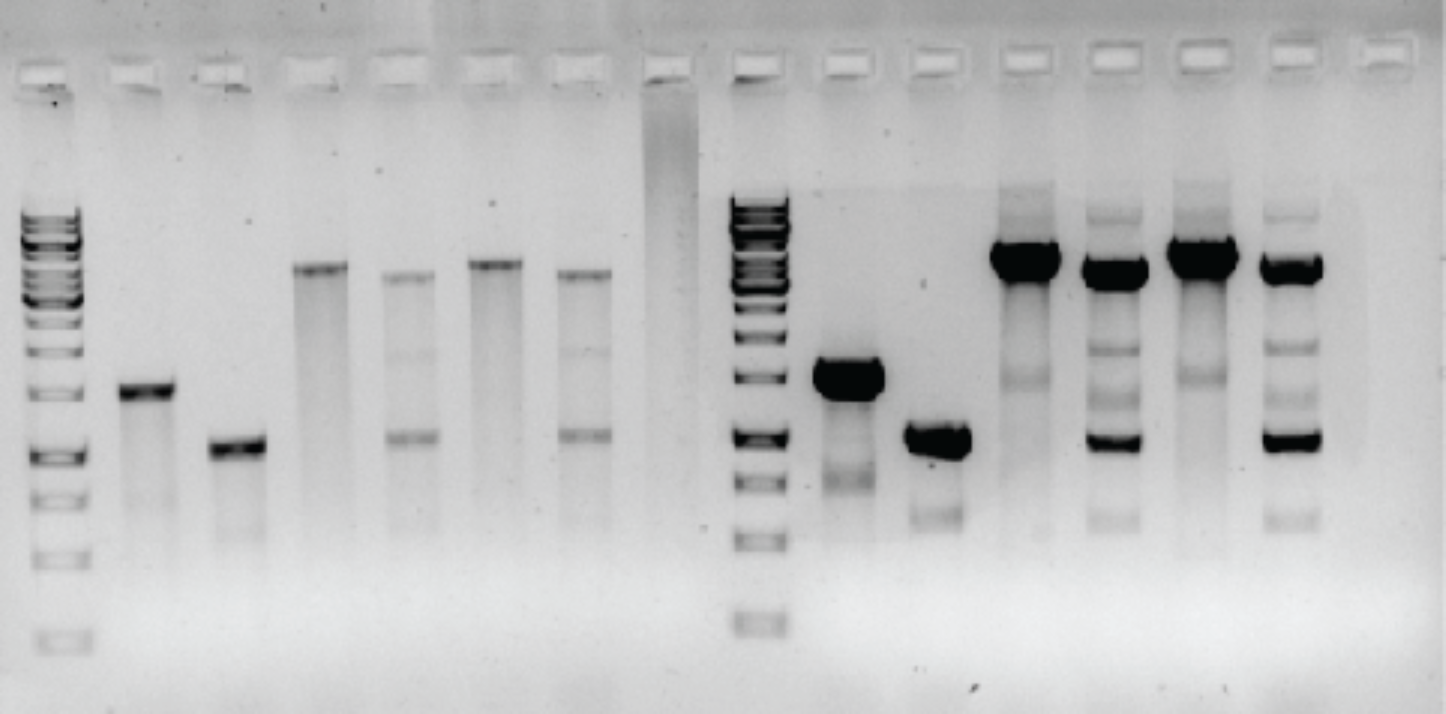

Supplement: Figure 4—figure supplement 1—source data 1. [file elife-104530-fig4-figsupp1-data1.zip › Figure4-S1-source data1/PCR uncroped no mark.png]

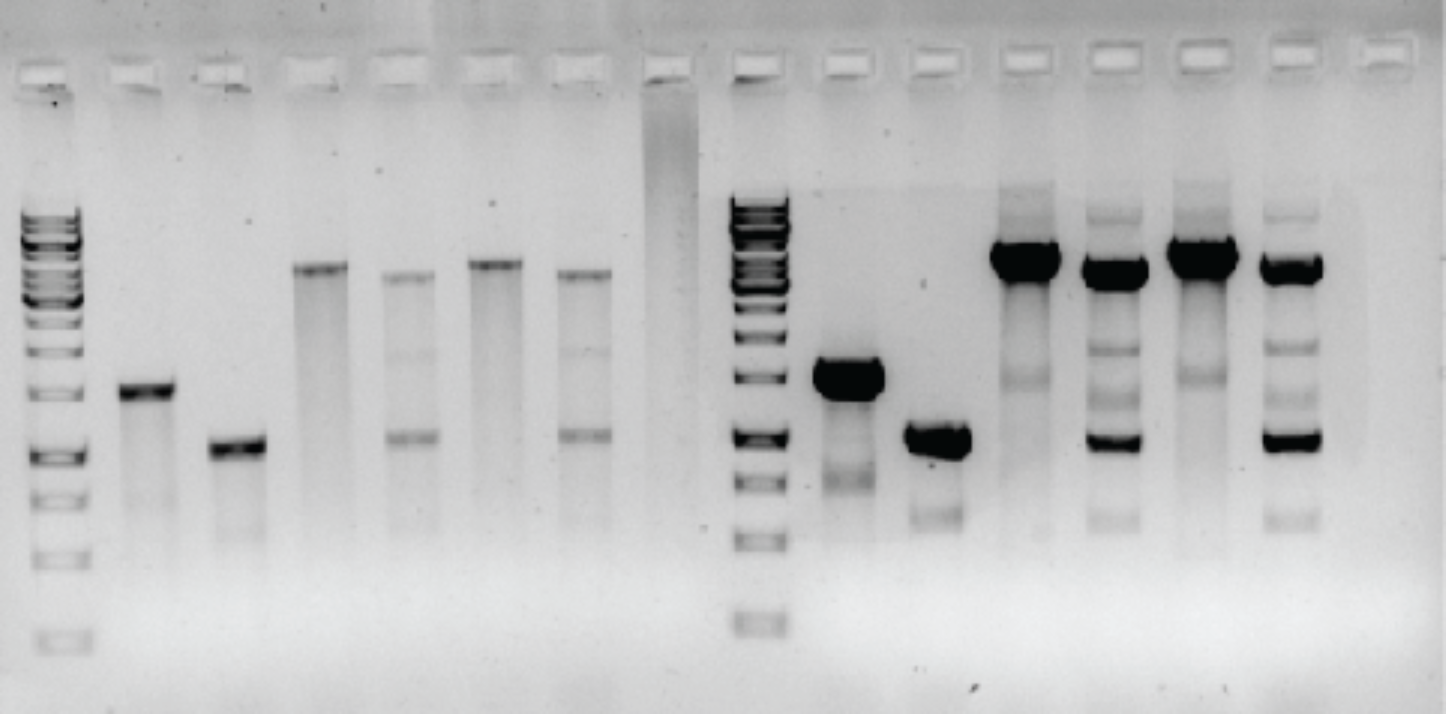

Supplement: Figure 4—figure supplement 1—source data 2. [file elife-104530-fig4-figsupp1-data2.zip › Figure4-S2-source data2/PCR labeled uncroped labeled.tif]

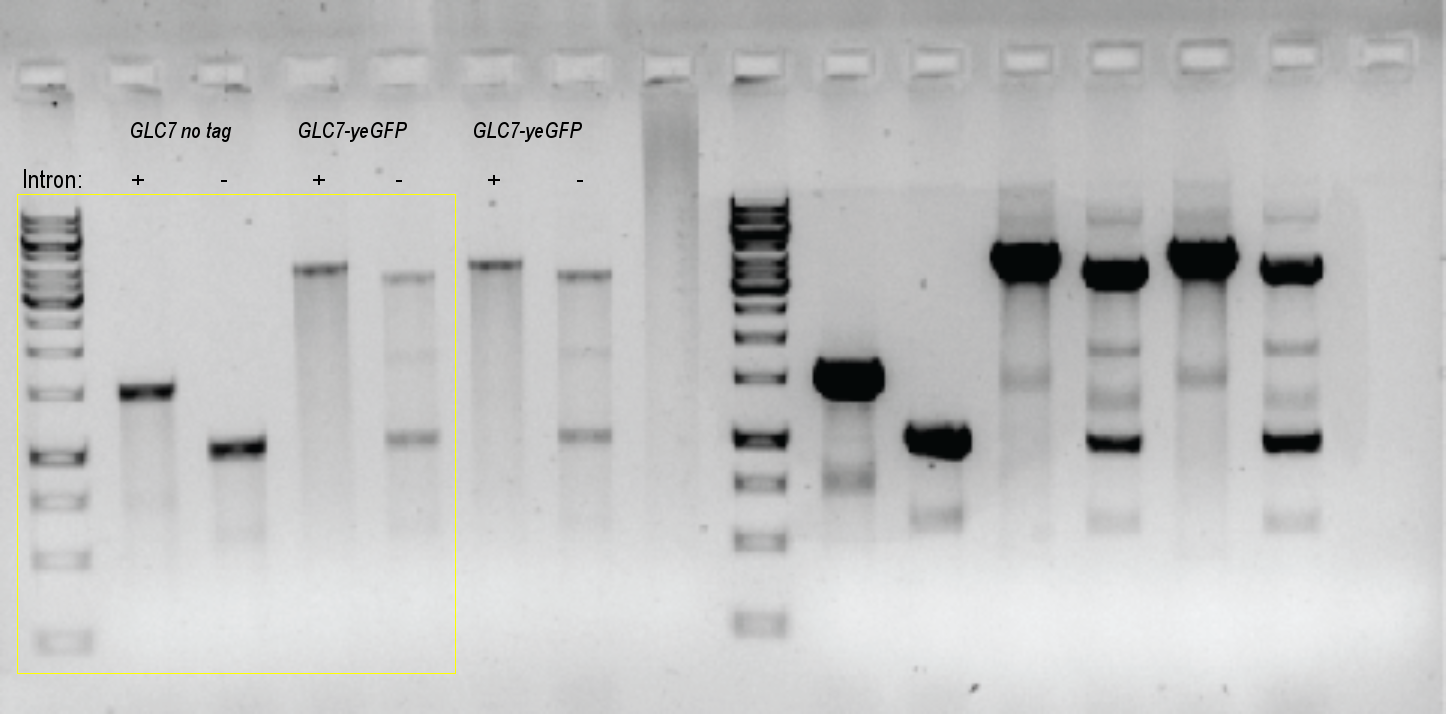

Supplement: Figure 4—figure supplement 1—source data 2. [file elife-104530-fig4-figsupp1-data2.zip › Figure4-S2-source data2/PCR labeled uncroped labeled.png]
