# Supplementary material for: Dissociation of the nuclear basket triggers chromosome loss in aging yeast
Source: eLife. 2025 Oct 30;14:RP104530. doi: 10.7554/eLife.104530 (PMC12574999; doi:10.7554/eLife.104530)
Supplement: Supplementary file 1. [file elife-104530-supp1.pdf]

# Yeast strain list:
# S288C background: ura3-52 his3Δ200 leu2 lys2-801 ade2-101 trp1Δ63

| YYB number | Genotype | Mating type | Plasmid | Source |
|---|---|---|---|---|
| 962 | wt deletion collection | A | | Deletion collection |
| 3416 | hoD::SCW11pr-Cre-EBD78-NatMX loxP-UBC9-loxP-LEU2 loxP-CDC20-Intron-loxP-HPHMX | α | | Dan Gottschling lab |
| 3476 | trp1::TetO:TRP1, lys4::LacO:LEU2 ,his3::LacR-GFP:HIS3 ,TetR-RFP | A | | Gabriel Neurohr |
| 14507 | LEU2::TetR-3mCherry POA1::TetO112:URA3 | A | | Karsten Weis lab(KWY3759) |
| 16094 | Spc42-mCherry:NAT leu2::LEU2 TetR-GFP | A | ec-URA3-CEN-rec-tetO-LEU | Anna Marzelliusadottir |
| 16128 | lys4::LacO:LEU2 his3::LacR-GFP:HIS3 Spc42-mCherry:NAT | α | | Anna Marzelliusadottir |
| 16157 | trp1::pRS405_Trp1_SplicingReporter | A | | Anne Meinema |
| 16161 | fob1::KAN, trp1::pRS405_Trp1_SplicingReporter | A | | Anne Meinema |
| 16301 | sir2::kanMX, trp1::pRS405_Trp1_SplicingReporter | A | | Anne Meinema |
| 16648 | mad1::kanMX | A | | Deletion collection |
| 16934 | lys4::LacO:LEU2 his3::LacR-GFP:HIS3 Spc42-mCherry:NAT | A | | Mihailo Mirkovic |
| 16935 | lys4::LacO:LEU2 his3::LacR-GFP:HIS3 Spc42-mCherry:NAT *Ipl1-321* | α | | Mihailo Mirkovic |
| 16936 | lys4::LacO:LEU2 his3::LacR-GFP:HIS3 Spc42-mCherry:NAT *GLC7-Δi* | A | | Mihailo Mirkovic |
| 16937 | lys4::LacO:LEU2 his3::LacR-GFP:HIS3 Spc42-mCherry:NAT Ipl1-321 *GLC7-Δi* | α | | Mihailo Mirkovic |
| 17198 | trp1::TetO:TRP1, lys4::LacO:LEU2 ,his3::LacR-GFP:HIS3 ,TetR-mRFP fob1::hphNT1 | A | | Mihailo Mirkovic |
| 17213 | mad2::KanMX | A | | Deletion collection |
| 17590 | wt elela intronless collection | A | PYB 2665 Ubi-GFP | Mihailo Mirkovic |
| 17591 | *GLC7Δi* | A | PYB 2665 Ubi-GFP | Mihailo Mirkovic |
| 17592 | *Ipl1-321* | α | PYB 2665 Ubi-GFP | Mihailo Mirkovic |
| 17593 | *Ipl1-321 GLC7Δi* | α | PYB 2665 Ubi-GFP | Mihailo Mirkovic |
| 17677 | POA1::TetO112:URA3 leu2::TetR-GFP:LEU2,Spc42-mCherry-hphNT1 | α | | Mihailo Mirkovic |
| 17678 | POA1::TetO112:URA3,leu2::TetR-GFP:LEU2,Spc42-mCherry-hphNT1 *GLC7Δi* | α | | Mihailo Mirkovic |
| 17679 | POA1::TetO112:URA3,leu2::TetR-GFP:LEU2,Spc42-mCherry-hphNT1 *GLC7/MCM21/NBL1 ΔiΔiΔi* | A | | Mihailo Mirkovic |
| 17689 | *Ipl1-321* | A | | Mihailo Mirkovic |
| 17690 | *Ipl1-321 GLC7Δi* | α | | Mihailo Mirkovic |
| 17691 | *Ipl1-321 MCM21/NBL1ΔiΔi* | A | | Mihailo Mirkovic |
| 17692 | *Ipl1-321 GLC7/MCM21/NBL1-ΔiΔiΔi* | A | | Mihailo Mirkovic |
| 17693 | wt Ipl1 crosses | α | | Mihailo Mirkovic |
| 17694 | *GLC7Δi* | A | | Mihailo Mirkovic |
| 17695 | *GLC7/MCM21/NBL1 ΔiΔiΔi* | A | | Mihailo Mirkovic |
| 17834 | POA1::TetO112:URA3 leu2::TetR-GFP:LEU2,Spc42-mCherry-hphNT1 fob1::Nat | α | | Mihailo Mirkovic |
| 17838 | POA1::TetO112:URA3 leu2::TetR-GFP:LEU2,Spc42-mCherry-hphNT1 sir2::Nat | α | | Mihailo Mirkovic |
| 17924 | Ipl1-321 Glc7Δi POA1::TetO112:URA3 leu2::TetR-GFP:LEU2 Spc42:mCherry:hphNT1 | α | | Mihailo Mirkovic |
| 18019 | POA1::TetO112:URA3 leu2::TetR-GFP:LEU2 Spc42-mCherry-hphNT | α | | Mihailo Mirkovic |
| 18028 | POA1::TetO112:URA3 leu2::TetR-GFP:LEU2 Spc42:mCherry:hphNT1 | α | | Mihailo Mirkovic |
| 18029 | Ipl1-321 POA1::TetO112:URA3 leu2::TetR-GFP:LEU2 Spc42:mCherry:hphNT | A | | Mihailo Mirkovic |
| 18069 | POA1::TetO112:URA3 leu2::TetR-GFP:LEU2 Spc42-mCherry-hphNT1 | α | | Jordan Mccarthy |
| 18070 | POA1::TetO112:URA3 leu2::TetR-GFP:LEU2 Spc42-mCherry-hphNT1 hrb1::kan gbp2::kan | α | | Jordan Mccarthy |
| 18071 | POA1::TetO112:URA3 leu2::TetR-GFP:LEU2 Spc42-mCherry-hphNT1 hrb1::kan gbp2::kan *GLC7/MCM21/NBL1-ΔiΔiΔi* | A | | Jordan Mccarthy |
| 18100 | POA1::TetO112:URA3 leu2::TetR-GFP:LEU2 Spc42-mCherry-hphNT1 *TUB1/TUB3-ΔiΔi* | A | | Mihailo Mirkovic |
| 18100 | POA1::TetO112:URA3 leu2::TetR-GFP:LEU2 Spc42-mCherry-hphNT1 *GLC7/MCM21/NBL1-ΔiΔiΔi* | A | | Mihailo Mirkovic |
| 18213 | POA1::TetO112:URA3 leu2::TetR-GFP:LEU2 Spc42-mCherry-hphNT1 snu66del::Nat | α | | Mihailo Mirkovic |
| 18214 | POA1::TetO112:URA3 leu2::TetR-GFP:LEU2 Spc42-mCherry-hphNT1 *TUB1/TUB3-ΔiΔi* snu66::Nat | α | | Mihailo Mirkovic |
| 18493 | POA1::TetO112:URA3 leu2::TetR-GFP:LEU2 Spc42-mCherry-hphNT1 sgf73::Nat | α | | Mihailo Mirkovic |
| 18661 | POA1::TetO112:URA3 leu2::TetR-GFP:LEU2 Spc42-mCherry-hphNT1 mlp1::nat | α | | Mihailo Mirkovic |
| 18663 | POA1::TetO112:URA3 leu2::TetR-GFP:LEU2 Spc42-mCherry-hphNT1 mlp1::nat , *GLC7/MCM21/NBL1-ΔiΔiΔi* | A | | Mihailo Mirkovic |
| 18683 | POA1::TetO112:URA3 leu2::TetR-GFP:LEU2 Spc42:mCherry:hphNT1 *NBL1Δi MCM21Δi* | A | | Mihailo Mirkovic |
| 19347 | POA1::TetO112:URA3 leu2::TetR-GFP:LEU2 Spc42-mCherry-hphNT1 *GLC7/MCM21/NBL1-ΔiΔiΔi* sir2::Nat | A | | Mihailo Mirkovic |
| 19348 | POA1::TetO112:URA3 leu2::TetR-GFP:LEU2 Spc42-mCherry-hphNT1 trp1::TetO:TRP1 | A | | Mihailo Mirkovic |
| 19453 | POA1::TetO112:URA3,leu2::TetR-GFP:LEU2,Spc42-mCherry-hphNT1 | α | YRp17(PYB546) | Mihailo Mirkovic |
| 19454 | POA1::TetO112:URA3,leu2::TetR-GFP:LEU2,Spc42-mCherry-hphNT1 *GLC7/MCM21/NBL1 ΔiΔiΔi* | A | YRp17(PYB546) | Mihailo Mirkovic |

# Primer list:

| Internal reference MM | Primer name | Primer Sequence | Source |
|---|---|---|---|
| 10 | 4RV_898hphNT1_HygroORF | CGG ATG TGA TGT GAG AAC TGT ATC | Mihailo Mirkovic |
| 36 | SPC42_Flanking_FW | CGT GTT ACA CAA AAG CGG TTT CG | Mihailo Mirkovic |
| 37 | SPC42_Flanking_RV | CGC TTT AAG AAT GCG CCA TAC TC | Mihailo Mirkovic |
| 38 | S1 Mlp1 Knopp | ACT CGC CGA AGC TAC ACA AAT AGT CAG TAA CGC CAC GTT TTA GGA TAA TGC GTA CGC TGC AGG TCG AC | Mihailo Mirkovic |
| 39 | S2 Mlp1 Knopp | TTG AAA AAG GTT TAG TTT GTA TTG ATC CCT TGT TTT TAC TAT CTC CTT TAA TCG ATG AAT TCG AGC TCG | Mihailo Mirkovic |
| 40 | Mlp1 50bp upstream FW | GCT GAC TAG GAC TTA ACT GAT ACT CG | Mihailo Mirkovic |
| 41 | Mlp1 RV ORF | CGG TTT TTT GTC TGT AGG AGA GAT AC | Mihailo Mirkovic |
| 64 | Glc7_flank_FW | GGT GAA CGA ACG AGT AAC TGG | Mihailo Mirkovic |
| 65 | Glc7_flank_RV | CCT TTT TAA ACT TTG ATT TAG GAC GTG | Mihailo Mirkovic |
| 97 | SIR2 flank FW | CAA ACC ATT TTT CCC TCA TCG GC | Mihailo Mirkovic |
| 98 | SIR2 flank RV | GGC GGT ACA TGT AAT ATT TCA CCC | Mihailo Mirkovic |
| 99 | SIR2_inORF_Fw | GAC CAT CCC ACA TAT GAA ATA CGC | Mihailo Mirkovic |
| 100 | SIR2_InORF_Rv | GGG TTT TGG GAT GTT CAT CTG ATG | Mihailo Mirkovic |
| 101 | Fob1_ORF_FW | CGA AAC CGC GTT ACA ATG ACG | Mihailo Mirkovic |
| 102 | Fob1_ORF_RV | GCC AAA GTC TCT TGT TTG ATC TCC | Mihailo Mirkovic |
| 103 | Fob1_upstream_FW | CGA TTG TGT GAG TGT GAA TTT GTG C | Mihailo Mirkovic |
| 104 | Fob1_downstream_RV | CCT TGC GGG CAA GAT CAT ATT ATC | Mihailo Mirkovic |
| 127 | HRB1_inORF_FW | CAG AAA ACG TAT GTT TCA TTG ATA AGG G- | Mihailo Mirkovic |
| 128 | HRB1_inORF_RV | CGT ACG ATA TAT CCA AAT CAC AAC CCC | Mihailo Mirkovic |
| 129 | HRB1_S1 | ACA GTT TGA ATG AGC AGA TAG AAC GCC AAA TAT TAA GTT AAC AGG ATA TGC GTA CGC TGC AGG TCG AC | Mihailo Mirkovic |
| 130 | HRB1_S2 | TTG TCG CAG ATC CAA TAG GTG AGA AAG TAT ATA GAT CGA GAG TAG TTC TAA TCG ATG AAT TCG AGC TCG | Mihailo Mirkovic |
| 132 | TUB1_Flank_Fw | CCC AAG ATC TGT AAA CTT ACA ACT GC | Mihailo Mirkovic |
| 133 | TUB1_Flank_Rv | CTT GAA ATA TAG AAA GGA TAA GGA GGT TGG | Mihailo Mirkovic |
| 134 | TUB3_Flank_Fw | CAT TAA TCG ACT AAG CAA GCG ACT TGA G | Mihailo Mirkovic |
| 135 | TUB3_Flank_Rv | GGT ATC GGG ATC AGA GCC ACT TTC | Mihailo Mirkovic |
| 145 | Tub1_Intron_FW_Check | CCC AAG ATC TGT AAA CTT ACA ACT GC | Mihailo Mirkovic |
| 146 | Tub1_Intron_RV_Check | GGA ATA TAA TTC CCA ACA GGC ATT AC | Mihailo Mirkovic |
| 147 | Tub3_Intron_FW_Check | GCG ACT TGA GAC AAT GAG AGA GG | Mihailo Mirkovic |
| 148 | Tub3_Intron_RV_Check | GCA TGC ATT ACC TAT TTG ACA ACC | Mihailo Mirkovic |
| 149 | Mcm21_Intron_FW_Check | CAG CAG GAC ATT GAA TCT TTA CTC AG | Mihailo Mirkovic |
| 150 | Mcm21_Intron_RV_Check | CGC TTT GAG CTT CTC ACG ACT TT | Mihailo Mirkovic |
| 151 | NBL1_Intron_FW_Check | CCG AAG AAC GGC AGA AGT TGC | Mihailo Mirkovic |
| 152 | NBL1_Intron_RV_Check | CCT CTC TGA TTA GTT CCT TTG ATT GAG | Mihailo Mirkovic |
| 256 | S1_Fob1 | GAA CAA TTT AAC GAT TGT GTG AGT GTG AAT TTG TGC TGA GGA TAA CAA TGC GTA CGC TGC AGG TCG AC | Mihailo Mirkovic |
| 257 | S2_Fob1 | TTT TTT CAC CTA TGG TGA CTC CTC CTT TCA TTC TAT CCT ACA TAT TAT TAA TCG ATG AAT TCG AGC TCG | Mihailo Mirkovic |
| 354 | S1_SNU66 | TTG ATA TTA ATA AAA AGG CAA TCA TCA CAT CAA CCC TTA ATT AAT TTA TGC GTA CGC TGC AGG TCG AC | Mihailo Mirkovic |
| 355 | S2_SNU66 | AGA TAT TGA ATA TTA AAA GCT GGG GCT AGT AGA ATT TGC TGA TTA GGT TAA TCG ATG AAT TCG AGC TCG | Mihailo Mirkovic |
| 356 | Fw_SNU66_Flank | GCG CAA CTT AAA ATT ATT CGC GGG | Mihailo Mirkovic |
| 357 | Rv_SNU66_Flank | GAA AAG TAC TTC CGA ATT CAA GGC C | Mihailo Mirkovic |
| 311 | Fw_1868_to 2640_Ubi-GFP_ADH_termina | CTT GTT TCT TTT TCT GCA CAA TAT TTC AAG CTA TAC CAA GCA TAC AAT CAA CTA TGC AGA TTT TCG TCA AGA CTT TGA CC | Mihailo Mirkovic |
| 312 | RV_1868_Ubi-GFP_to MscI SacII_PYB26 | AGC TCT GAT AGA GTT GGT CAA GAC CAA TGC GGA GCA TAT ACG CCC GGA GCC GCG GCC GGT AGA GGT GTG GTC AAT AAG AG | Mihailo Mirkovic |
| 389 | sgf73_inORF_FW | GTA GAC CAC TTA GAA AAC CAT TGT GC | Mihailo Mirkovic |
| 390 | sgf73_inORF_RV | CGT CAT GAG GTT ATT GTT ACC GTT TT | Mihailo Mirkovic |
| 391 | MCM21_inORF_FW | CTC AGT GAA AGT ATG TGA TTT TGA GGC | Mihailo Mirkovic |
| 392 | MCM21_inORF_RV | CCA AAT CAT CCA AAG AAC CGA GC | Mihailo Mirkovic |
| 751 | S1_Sir2 | CGGTAGACACATTCAAACCATTTTTCCCTCATCGGCACATTAAAGCTGGA TGcgtacgctgcaggtcgac | Annina Denoth-Lipp |
| 752 | S2_Sir2 | GATATTAATTTGGCACTTTTAAATTATTAAATTGCCTTCTACTTAatcga tgaattcgagctcg | Annina Denoth-Lipp |

# RNA FISH-Stellaris probes:

| Cal Fluor Red 590 | | Cal Fluor Red 590 | | Cal Fluor Red 590 | |
|---|---|---|---|---|---|
| **DBP2 intron probes** | **Probe number** | **YRA1 intron probes** | **Probe number** | **GLC7 intron probes** | **Probe number** |
| Probe sequence 5' to 3' | | Probe sequence 5' to 3' | | Probe sequence 5' to 3' | |
| ctgtaatatcgttaggggtaac | DBP2_1 | acgagacgatgcgagtaactaa | YRA1_1 | gcgaagttcaacattaacatac | GLC7_1 |
| atgtcattaaaagccattagga | DBP2_2 | catatccttccttacaaagaat | YRA1_2 | ctctaggcattctatctcttga | GLC7_2 |
| tcatggttgtcataaagtcatt | DBP2_3 | gaattcttgatcaaaagcgtgt | YRA1_3 | gtagaacacataaaacacctga | GLC7_3 |
| aaaaaggtctctcttctgtact | DBP2_4 | ggactctttcttcatctctaaa | YRA1_4 | aactatcttactgccgcaacag | GLC7_4 |
| gtctagattcgaaaaggctcaa | DBP2_5 | ttgtattttctctagcgaagta | YRA1_5 | cagtcaaacggaaataactgct | GLC7_5 |
| catcgagaatagactcacatag | DBP2_6 | ggtatggttggcttatgaaaac | YRA1_6 | catcagatgcttggtaaattct | GLC7_6 |
| aaaattcaatttccgatactcc | DBP2_7 | ggccatgatttgaagtattgaa | YRA1_7 | tatctcgacatatgagtaggct | GLC7_7 |
| atgcattagaagtcattcgaat | DBP2_8 | tagctggtaattccaaccttat | YRA1_8 | gctaaacacacacattatctcg | GLC7_8 |
| cccaatattagttttctgtagt | DBP2_9 | aagaacaaacccattctcagtg | YRA1_9 | gccggatgatgatacaaaacat | GLC7_9 |
| ttaaagtcaatttttctcatcc | DBP2_10 | agattgatttgagttaacccag | YRA1_10 | ggatttttctgaatatgggagg | GLC7_10 |
| tataatttcccgtctctcaaaa | DBP2_11 | gattgacagattagcagatagt | YRA1_11 | cttcttttttagtgtgagcaa | GLC7_11 |
| gaacacaaatacacttctccga | DBP2_12 | gacacaatacgccagcttaaaa | YRA1_12 | agattcgttttcttcataacga | GLC7_12 |
| cttttgacatgaagtgcaaatc | DBP2_13 | taggaactaactatcacggtcg | YRA1_13 | ctttctctcgggctcaaaaaaa | GLC7_13 |
| gccgatgtcaaaaagcgaaatt | DBP2_14 | agtattggatgatacgcttatt | YRA1_14 | aaatccacatttcttggttttc | GLC7_14 |
| aagccaattccttgtttatttg | DBP2_15 | ttaattgatccttccacaactt | YRA1_15 | tgctgtcaaaaacgcttcattt | GLC7_15 |
| atgcgttctagaaataagctga | DBP2_16 | ctccctcttgaaaggatacaaa | YRA1_16 | ccgttgactttaagagaacaat | GLC7_16 |
| acaacgatcaacgaagcgtatg | DBP2_17 | tggtgccattcgaaactgaaag | YRA1_17 | atttgtcactaaatctagctca | GLC7_17 |
| gtacaaatgccaagcattgaaa | DBP2_18 | ttcccatagctattttttgaaa | YRA1_18 | | |
| ttccaataaaacaatctaccct | DBP2_19 | tttcgaacgttcatgaaagaca | YRA1_19 | | |
| gatgacggcatttcaaagtaga | DBP2_20 | aatgaagcgatgcactcatatc | YRA1_20 | | |
| ggatgcaacagtatctcaatat | DBP2_21 | gtttcattaggtgtctcaaatc | YRA1_21 | | |
| tcagcacttcttaataattcga | DBP2_22 | cctcaaataaacatgtaccacg | YRA1_22 | | |
| ttattttgttattttccatttt | DBP2_23 | aagtataccattgcgtaggaaa | YRA1_23 | | |
| ttctgaatatggttgaaatcct | DBP2_24 | acaatcaaggagttgagagtat | YRA1_24 | | |
| actgaatgaaagctagcactta | DBP2_25 | aagaaggaagggacaatacaca | YRA1_25 | | |
| atcaatactccagaatccttat | DBP2_26 | | | | |
| ggcttgaagattatcagaaatt | DBP2_27 | | | | |
| caacattgtctcaaatttcccc | DBP2_28 | | | | |
| ccacaaattcatgtttggactc | DBP2_29 | | | | |
| tatgaactttattttcatgccc | DBP2_30 | | | | |
| tactgttagtaatttggttcgt | DBP2_31 | | | | |
